# Gamification and motivation in adolescents. Systematic review from Physical Education

**DOI:** 10.3389/fpsyg.2025.1575104

**Published:** 2025-03-24

**Authors:** Alejandro Sal-de-Rellán, Álvaro Hernández-Suárez, Ariadna Hernaiz-Sánchez

**Affiliations:** Department of Education and Educational Innovation, Faculty of Law, Education and Humanities, Universidad Europea de Madrid, Madrid, Spain

**Keywords:** motivation, gamification, physical education, adolescents, engagement, learning outcomes, pedagogical innovation

## Abstract

**Introduction:**

Physical Education plays a crucial role in adolescent health, but motivation remains a challenge as participation declines during this stage. Gamification, which integrates game elements into learning, has gained attention as a methodology to enhance it. However, its effectiveness in Physical Education requires further exploration.

**Methods:**

This systematic review followed PRISMA guidelines. A search was conducted in Dialnet, PubMed, ERIC, Scopus, and Web of Science for studies published between 2015 and January 2025. Research focusing on gamification and motivation in secondary and high school Physical Education was selected based on predefined criteria. The methodological quality was assessed using the PEDro scale.

**Results:**

A total of 19 studies met the inclusion criteria. The findings indicate that gamification enhances motivation in Physical Education, whether applied independently or combined with other methodologies. Additional benefits include improved autonomy, social skills, and classroom atmosphere. However, its impact on academic performance and motor skill development remains inconclusive. One study reported potential drawbacks when gamification neglects affective-motivational skills.

**Discussion:**

Gamification appears to be an effective tool for increasing motivation in Physical Education. However, methodological inconsistencies limit the generalizability of results. Future research should include control groups, clearer methodologies, and long-term evaluations to assess its sustained impact.

## 1 Introduction

Physical and sports activities have a positive influence on an individual’s health (Martins et al., 2018). For this reason, Physical Education plays a fundamental role in adolescence, as a large proportion of adolescents only come into contact with Physical Education through this subject (Hernaiz-Sánchez and Bäder-Gilabert, 2023).

During adolescence, a number of changes take place in the body, physically, emotionally and socially (Ortega et al., 2007), making this stage a challenge for all teachers in general and for Physical Education teachers in particular, as this is the stage in which a large proportion of pupils drop out of physical and sporting activities (Aznar-Ballesta and Vernetta, 2023). This subject is not only intended to value health but also seeks the well-being of students by promoting improvements at social, emotional and cognitive levels (Bailey, 2006). In this sense, Physical Education helps to develop motor skills, coordination, flexibility and endurance, contributing to an active and healthy lifestyle (Strong et al., 2005). It also promotes body awareness and self-esteem, allowing teenagers to explore and understand their physical capabilities (Trudeau and Shephard, 2008). Physical Education lessons also provide opportunities for teamwork, as well as social and physical interaction with peers (Bailey, 2006). This helps them understand the importance of respecting the limitations of others, while fostering empathy and inclusion in and out of the classroom (Bailey, 2006). In addition, some authors point out that physical activity during school hours contributes to combating stress and helps concentration, simultaneously improving focus and health (Strong et al., 2005). For these reasons, it plays an essential role in the holistic development of adolescents and contributes to their transformation into well-balanced and healthy individuals (Ortega et al., 2007). Furthermore, some sources suggest that the improvement of students’ mood contributes to adherence to physical and sporting activity both inside and outside the classroom (Trudeau and Shephard, 2008).

Commitment to the practice of physical and sports activities is one of the great challenges of Physical Education. In this respect, several studies point to the fact that greater motivation and enjoyment of the subject will improve students’ engagement and extracurricular practice (Biddle et al., 2004; Haerens et al., 2010; Comte et al., 2015; Aznar-Ballesta and Vernetta, 2023). For this reason, motivation towards the subject plays a crucial role in promoting long-term healthy lifestyles (Calogiuri, 2016). On this matter, studies have shown that more entertaining Physical Education sessions will lead to a better attitude towards them (Gómez Mármol et al., 2015) and, consequently, a better adherence to healthy lifestyles.

Given the importance of Physical Education for the integral development of students, various learning methods have been developed to improve their motivation towards the subject, so that learning is as meaningful as possible and influences their extracurricular habits. Said motivation may be generated through the enjoyment of the subject itself, through the achievement of small and long-term objectives or even through the very methodology presented by the teacher (Vasconcellos et al., 2020). In order for student motivation to be constant over time, it is important to generate engaging and meaningful environments that develop students’ intrinsic motivation towards the subject not only for the rewards obtained (Ryan and Deci, 2020). To achieve positive motivational environments there is a variety of techniques, activities, proposals and pedagogical models (Standage et al., 2003). Gamification is one of the proposals that is being carried out in classrooms to increase motivation (El-Tanahi et al., 2023; Ferriz-Valero et al., 2023; Sotos-Martínez et al., 2023, 2024). According to Marín (2018), gamification arises from the observation of the success of video games and is proposed as the application of a methodology that establishes an educational strategy based, on a technique of rewards and incentives and, in addition, on an integration of the principles of video games trying to create attractive and effective learning experiences that are engaging and motivating for the students (Dichev and Dicheva, 2017; Fernandez-Rio et al., 2020). Moreover, some studies suggest that devoting time and focus to achieving such rewards and rewards improves concentration as well as physical and mental effort (Zichermann and Cunningham, 2011). This approach not only makes sessions more fun and engaging but it can also offer small incentives that maintain student interest and engagement (Pourabbasi et al., 2020).

Given the positive reception by students and the influence on increasing motivation, it seems that gamification may be an effective tool to promote healthy habits and combat physical inactivity (Landers and Landers, 2015). For this reason, it seems that it not only improves academic performance, but also promotes a balanced and healthy lifestyle, reinforcing positive behaviours that can extend beyond the classroom (Monguillot Hernando et al., 2015; Fernandez-Rio et al., 2021). Based on the positive results derived from its use, an increasing number of teachers and researchers are including gamification in their teaching practices and research studies (Rodríguez-Escaravajal and Martín-Acosta, 2019).

Among the main positive outcomes that gamification brings to Physical Education, the increase of students’ motivation in Physical Education classes must be emphasised, which makes participation more active and sustained. It is clear that achievements and rewards can positively influence engagement (Real-Pérez et al., 2021; Serrano-Durá et al., 2021; Hernández-Rubio et al., 2023). In addition to increased motivation, other authors highlight the development of motor skills, examining how games can enhance their learning and practice in physical activities, and testing the development of specific skills such as coordination, balance and basic skills (Sevilla-Sanchez et al., 2023).

In relation to other aspects associated with integral education, there are authors who point out improvements and benefits related to collaboration and teamwork. They further analyse how gamification can improve these aspects in educational environments by boosting cooperation among students and regenerating social skills (Melero et al., 2022).

From a more global point of view, other authors state that gamification is presented as an effective methodology to improve the external regulation and the general performance of students in the subject (Rouissi et al., 2020). According to authors such as Quintero González et al. (2018), gamification is a tool for improving student motivation by creating a playful and pleasant environment.

As a whole, the good use of gamification seems to be able to improve learning outcomes under different conditions and to influence the betterment of more autonomous study, increasing motivation and promoting more meaningful assessment practices for students (Godoy, 2019). Although it seems that the benefits of gamification have been observed in different populations, several studies point to a need for further research in this field in order to draw more methodologically rigorous conclusions (Geelan et al., 2015; Ferriz-Valero et al., 2023; Sotos-Martínez et al., 2024). Moreover, currently, no systematic reviews have been found that focus solely on the adolescent population. For this reason, the main objective of this study was to analyse the benefits of gamification proposals in Physical Education in relation to motivation and meaningful learning in adolescents.

## 2 Methods

### 2.1 Study design

In conducting this systematic review, the authors followed the Preferred Reporting Items for Systematic Reviews and Meta-Analyses (PRISMA) guidelines (Page et al., 2021).

### 2.2 Search strategy

A systematic search of five databases (Dialnet, Pubmed, Eric, Scopus and Web of Science) was conducted to identify articles published prior to 23 January 2025. Following the PICO (Population, Intervention, Comparison, Outcome) design provided by PRISMA (Table 1), the following search strategy was used to look for relevant articles, where the authors were not blinded to journal names or manuscripts’ authors: Gamification AND (“Physical education”) AND Motivation. Additionally, the reference lists of the studies retrieved were manually inspected to identify potentially eligible studies not captured by electronic means.

**Table 1:** Overview of PICO.

Element	Description
P (Population/Participants)	Secondary and high school students. Studies with kindergarten, primary or elementary school, or university students are excluded.
I (Intervention)	Use of gamification as a pedagogical strategy in physical education.
C (Comparison)	No specific comparison conditions are defined in the inclusion criteria. However, some studies may compare gamification with traditional teaching methods or other methodological approaches in physical education.
O (Outcome/Result)	Student motivation levels in physical education after implementing gamification.

### 2.3 Screening strategy and study selection

When the referred authors had completed the search (A.S-d-R., AL.H.S. and AR.H.S.), they compared their results to ensure that the same articles were identified. Then, one of the authors (A.S-d-R.) downloaded and copied the main data from the articles (title, authors, date, and database) onto an Excel spreadsheet (Microsoft Excel, Microsoft, Redmond, USA). Then, two authors (A.S-d-R. and AL.H.S.) removed duplicates. The remaining articles were screened and checked by two authors independently (A.S-d-R. and AR.H.S.) following the inclusion and exclusion criteria. Moreover, relevant articles not previously identified were also screened in an identical manner and further studies that complied with the inclusion–exclusion criteria were included and labelled as ‘included from external sources’.

### 2.4 Inclusion and exclusion criteria

Studies were included in the systematic review if they met the following criteria:

**Inclusion Criteria 1**: published in a peer-reviewed journal between 2015 and 2025. The last 10 years were selected in order to find the most recent gamified proposals.

**Inclusion Criteria 2**: focusing on secondary and high school students.

**Inclusion Criteria 3**: focusing on student motivation though Gamification or hybridization of models with Gamification.

**Inclusion Criteria 4**: written in English or Spanish.

Studies were excluded if:

**Exclusion Criteria 1**: they were not published in a peer-reviewed journal or were published outside the specified date range.

**Exclusion Criteria 2**: they were not an empirical research, i.e., research other than quantitative, qualitative, and mixed methods studies.

**Exclusion Criteria 3**: they focused on kindergarten, primary or elementary school, or University.

**Exclusion Criteria 4**: they were not written in English or Spanish.

### 2.5 Assessment of study methodology

The Physiotherapy Evidence Database (PEDro) scale was used to assess the methodological quality of pre-test and post-test studies with randomly selected experimental (EXP) and control (CON) groups. The scale scored the internal study validity in a range from zero (low methodological quality) to 10 (high methodological quality). The score that each section received ranged from zero (“no”) to one (“yes”), depending on the quality obtained by each point. Ten items were measured in the scale. Studies that scored from 9 to 10 on the PEDro scale were considered to be of excellent methodological quality. Studies with a score between six and eight have good methodological quality; between four and five, fair quality; and below four points, poor methodological quality (Maher et al., 2003).

## 3 Results

### 3.1 Study slection

**Figure 1:**
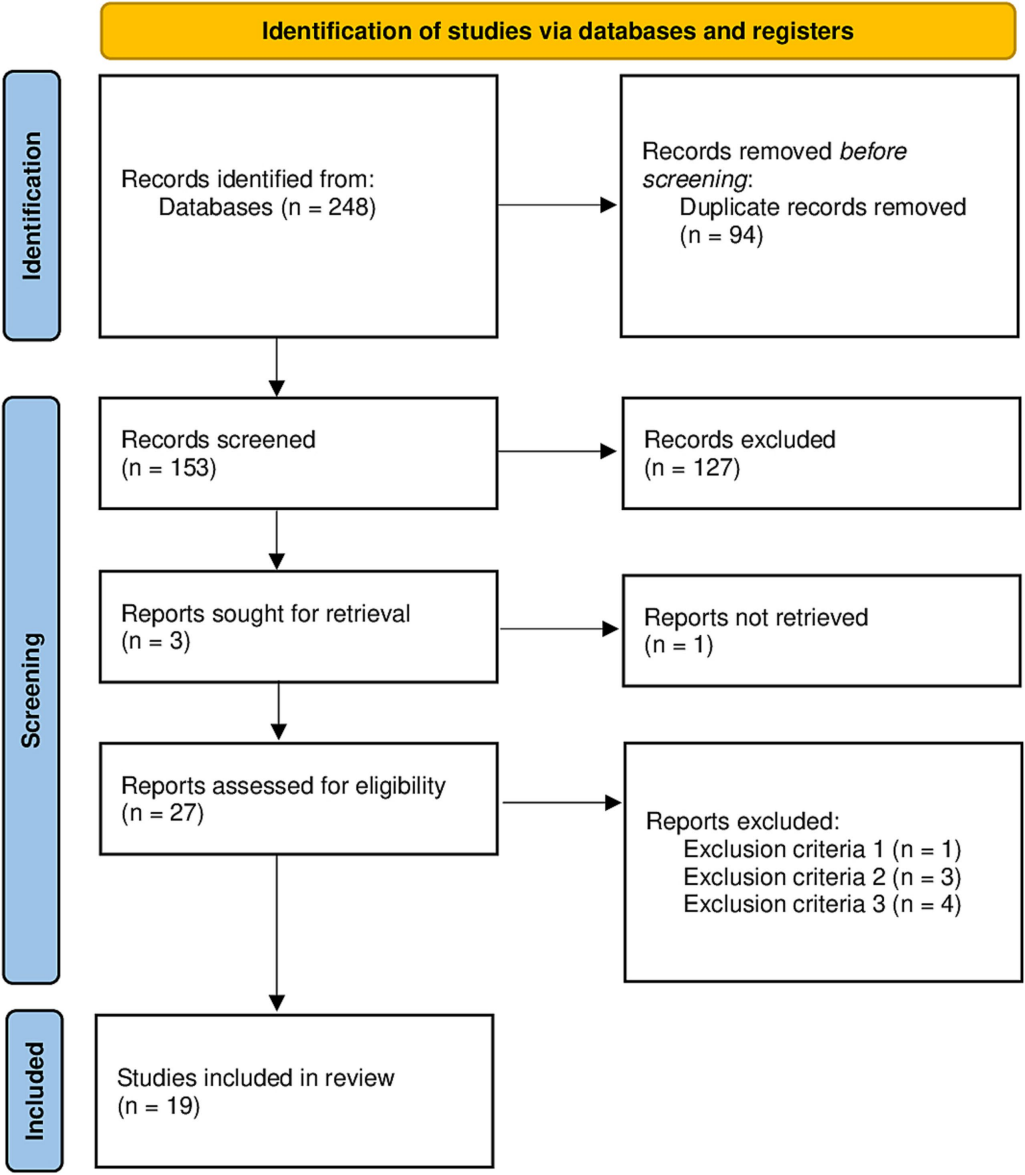
Flow diagram of the study.

A total of 248 (i.e., Dialnet: 55; Pubmed: 18; Eric: 13; Scopus: 77; and Web of Science: 85) original articles were initially retrieved from the mentioned databases, of which 94 were duplicates. Thus, a total of 153 original articles were found. After this, a total of 127 articles checked by title and abstract were excluded as they did not meet the inclusion criteria. The remaining 26 articles were checked in full, leading to the exclusion of one according to criterion n° 1, three according to criterion n° 2, and four according to criterion n° 3. A total of 19 articles met all the inclusion criteria and were finally considered in the qualitative synthesis. All the steps followed for the selection of the articles is available in Figure 1.

### 3.2 Quality assessment

To understand these results, it should be taken into consideration that many of the studies analyzed do not follow a methodology with a control group and an experimental group. For this reason, the scores could be low. The overall methodological quality of the cross-sectional studies can be found in Table 2.

**Table 2:** Methodological assessment of the included studies.

Reference	1	2	3	4	5	6	7	8	9	10	Score
Carrasco-Ramírez et al. (2019)	0	0	1	0	0	1	1	1	1	1	6
Fernandez-Rio et al. (2021)	1	1	1	0	0	1	1	1	1	1	8
Fernández-Vázquez et al. (2024)	1	1	1	0	0	1	1	1	1	1	8
Flores-Aguilar et al. (2023)	1	1	1	0	0	1	1	1	1	1	8
Lamoneda et al. (2022)	0	0	0	0	0	1	1	1	1	1	5
López-Urán et al. (2022)	0	0	1	0	0	1	1	1	1	1	6
Jiménez-Parra et al. (2023)	1	1	1	0	0	1	1	1	1	1	8
Martín-Moya et al. (2018)	0	0	1	0	0	1	1	1	1	1	6
Monguillot Hernando et al. (2015)	0	0	1	0	0	1	1	1	1	1	6
Navalón Almedros et al. (2022)	0	0	1	0	0	1	1	1	1	1	6
Ortega and Chacón-Borrego (2021)	0	0	1	0	0	0	0	1	1	1	4
Quintero González et al. (2018)	0	0	0	0	0	0	0	1	1	1	3
Real-Pérez et al. (2021)	1	1	1	0	0	1	1	1	1	1	8
Roure and Pasco (2023)	1	1	1	0	0	1	1	1	1	1	8
Segura-Robles et al. (2020)	1	1	1	0	0	1	1	1	1	1	8
Soriano-Pascual et al. (2022)	1	1	1	0	0	1	1	1	1	1	8
Sotos-Martínez et al. (2024)	1	1	1	0	0	1	1	1	1	1	8
Tenelema-Martínez and Loaiza-Dávila (2023)	1	1	1	0	0	1	1	1	1	1	8
Valero-Valenzuela et al. (2020)	1	1	1	0	0	1	1	1	1	1	8

Item 1 = subjects were randomly allocated to groups (in a crossover study, subjects were randomly allocated an order in which treatments were received); Item 2 = allocation was concealed; Item 3 = the groups were similar at baseline regarding the most important prognostic indicators; Item 4 = there was blinding of all subjects; Item 5 = there was blinding of all therapists who administered the therapy; Item 6 = there was blinding of all assessors who measured at least one key outcome; Item 7 = measures of at least one key outcome were obtained from more than 85% of the subjects initially allocated to groups; Item 8 = all subjects for whom outcome measures were available received the treatment or control condition as allocated or, where this was not the case, data for at least one key outcome was analyzed by “intention to treat”: Item 9 = the results of between-group statistical comparisons are reported for at least one key outcome; Item 10 = the study provides both point measures and measures of variability for at least one key outcome.

Out of the 19 included articles, none scored excellent methodological quality. Sixteen studies obtained a good methodological quality score (Monguillot Hernando et al., 2015; Martín-Moya et al., 2018; Carrasco-Ramírez et al., 2019; Segura-Robles et al., 2020; Valero-Valenzuela et al., 2020; Fernandez-Rio et al., 2021; Real-Pérez et al., 2021; López-Urán et al., 2022; Navalón Almedros et al., 2022; Soriano-Pascual et al., 2022; Flores-Aguilar et al., 2023; Jiménez-Parra et al., 2023; Roure and Pasco, 2023; Tenelema-Martínez and Loaiza-Dávila, 2023; Fernández-Vázquez et al., 2024; Sotos-Martínez et al., 2024). Finally, two studies with fair methodological quality (Ortega and Chacón-Borrego, 2021; Lamoneda et al., 2022) and one with poor methodological quality were included (Quintero González et al., 2018).

### 3.3 Individual results

The characteristics of the studies were extracted and are shown in Table 3.

**Table 3:** Effects of gamification on motivation in Physical Education lessons.

Reference	Aim	Sample	Study type	Intervention methodology	Results	Conclusion
Carrasco-Ramírez et al. (2019)	To compare the effects of GAM in comparison with TRM, regarding motivation and academic performance using the PE subject, also the teacher’s perception to find out the opinion and the formation that they have about it.	90 students (aged 17–18 years). EXP (*n* = 50), CON (*n* = 40)	Mixed methodology. An investigation-action	GAM vs. TRM	GAM obtained better results than TRM. Both methodologies were positively perceived by students. As regards the motivation, no significant differences were found between both methodologies. GAM consolidates as a good methodology to improve the academic results in PE. The use of different methodologies increases the motivation of the students.	GAM is a good strategy to improve academic performance. No significant differences were found as regards the motivation of the students during PE sessions.
Fernandez-Rio et al. (2021)	To compare the possible effects of GAM and TRM in secondary PE as regards intrinsic motivation, autonomy satisfaction, competence satisfaction, relatedness satisfaction, and intention to be physically active	54 students (14 ± 0.1 years, 26 girls and 28 boys, aged 14–15 years). EXP (*n* = 27, 13 boys, 14 girls), CON (*n* = 27, 15 boys, 12 girls)	Pretest, post-test quasi-experimental research design	GAM vs. TRM	Significant differences at post-tests favouring EXP in all the variables assessed. To use GAM in PE since it was associated with increased levels of students’ intrinsic motivation, basic psychological needs and intention to be physically active compared to TRM.	GAM could be considered a positive pedagogical framework for secondary PE increasing levels of students’ intrinsic motivation, basic psychological needs and intention to be physically active.
Fernández-Vázquez et al. (2024)	To examine the impact of combining VR and GAM with PTS on students’ motor skills and perceived effort in PE.	75 students (13.58 ± 0.68 years, 53.3% girls and 46.7% boys, aged 12–17 years). EXP 1 (*n* = 32), EXP 2 (*n* = 29), CON (*n* = 14)	Mixed-method study	PTS vs. PTS + GAM vs. PTS + GAM + VR	The PTS group reported a higher perceived effort compared to the other groups (*p* < 0.001). All study groups exhibited improvements in handgrip strength (*p* < 0.001) and flamingo (*p* < 0.05) tests, while lateral jump test improvements were observed only in the two GAM groups (*p* < 0.001). The VR group showed an improvement in the plate-tapping tests (*p* < 0.001), while the PTS group exhibited a decline in the displacement with support test (*p* < 0.05). Participants’ perceptions suggest that the activity nature, motivation from competition and rewards influenced perceived effort and motor skills.	GAM techniques are effective in reducing perceived effort in PE programmes and increasing motivation. Combining GAM with VR enhances improvement in motor skills.
Flores-Aguilar et al. (2023)	To analyse the impact on the motivational regulations and grades of secondary school students of GAM in PE classes compared to TRM	102 students (16.7 ± 0.43 years old, 64 girls and 38 boys, aged 15–17 years). EXP (*n* = 51, 18 boys and 33 girls), CON (*n* = 51, 20 boys and 31 girls)	Quasi-experimental design, pretest post-test	GAM vs. TRM	Only GAM achieved significant changes in intrinsic motivation, demotivation and identified, introjected and external regulations, although to a greater extent in intrinsic motivation. These students also obtained significantly higher final grades.	The application of GAM as an emerging pedagogical model can generate positive effects among students in terms of motivation and academic performance.
Jiménez-Parra et al. (2023)	To assess the impact of a PE teaching unit on PE satisfaction, basic psychological needs satisfaction, motivation, school social atmosphere and cognitive performance.	120 students (13,48 ± 1,36 years, 61 girls and 59 boys, aged 12–17 years). EXP (*n* = 61, 27 boys and 34 girls), CON (*n* = 59, 32 boys and 27 girls)	Quasi-experimental design	Hybridization (GAM + CL)	The programme showed improvements in the psychological mediator’s index, executive function of planning, PE students’ satisfaction and the school social atmosphere, which would make it suitable for the improvement in the performance of PE teachers in educational centres.	GAM – CL may be adequate to improve the degree of satisfaction of students towards PE. The hybridization of strategies implied a decrease in boredom, a greater satisfaction of basic psychological needs, more favourable school atmosphere and an improvement in the students’ executive planning function.
Lamoneda et al. (2022)	To describe a game-based educational intervention (GAM) on the literary work “The Alchemist” for the teaching of orienteering content, and to explore participants’ perceptions as well as assessing development during and after their participation in the intervention.	94 students (16.06 ± 0.73 years, 56,47% girls and 43,53% boys, aged 16–17 years)	Qualitative methodology through evaluative narrative research	Only GAM	The programme helped them to improve their social relationships, was a fun experience, and allowed them to wind down and to have a sense of freedom in nature.	GAM enhances student motivation, the interest in providing real learning experiences that lead to improved social relationships and personal life issues. Learning is meaningful and promotes education in personal, social and environmental values, in line with the promotion of SDG. The holistic development of pupils is encouraged from the point of view of acquiring knowledge mainly in the physical, social and affective domains.
López-Urán et al. (2022)	To analyze the self-determined motivation in two groups that received the same didactic unit of jump ropes, but with two different methodologies	118 students (14.04 ± 0.83, 84 girls and 98 boys, aged 12–16 years). The EXP 1 (*n* = 74, 35 boys and 39 girls), EXP 2 (*n* = 44, 29 boys and 15 girls)	Quasi-experimental quantitative methodology with cluster sampling design	GAM vs. Attitudinal style	A non-significant increase in motivation was observed in both groups.	The use of GAM, ICTs and additional prizes or badges increases motivation more than the mere use of “badges.” In the attitudinal style model, using a task assignment style can be counterproductive because it does not allow students to work on skills such as affective-motivational ones, essential to developing the social dimension proposed by SDG.
Martín-Moya et al. (2018)	To identify changes in the motivational dimensions of goal theory in Secondary School students, through an innovative intervention called “DiverHealth”	30 students (17–18 years, 15 girls and 15 boys, aged 17–18 years)	Quasi-experimental design with an EXP, taking Pretest and Post-test measures. The methodology used is quantitative descriptive.	Only GAM	Perception of self-perceived motor competence improved in the total-group (*p* < 0.003) and, especially, in the female-group (*p* < 0.011). Perception of compared motor competence improved in the total-group (*p* < 0.042). Learning commitment improved in both the total-group and the male-group (*p* < 0.01). Anxiety and fear of failure increased in the total-group (*p* < 0.007) and the female-group (*p* < 0.024)	An intervention based on physical activity and healthy habits learning through GAM could be useful for increasing the students’ motivation.
Monguillot Hernando et al. (2015)	To show the impact of the use of GAM as a learning strategy in PE for the development of healthy behaviours.	99 students (aged 13–14 years)	Qualitative socio-critical methodological perspective. Action-research design.	Only GAM	77% of pupils valued GAM as a motivational strategy for learning healthy behaviours and 98% stated that they had learned to apply the healthy heart rate. As for the use of ICTs, 84% of pupils learnt to use different tools such as prezi, voki, movie maker, QR codes. 75% of pupils obtained a mark of seven or more for the unit, which means that the unit’s objectives were largely achieved	GAM is an emerging learning strategy in PE to increase learning motivation and develop healthy habits in students.
Navalón Almedros et al. (2022)	To analyze which teaching style is most appropriate for the motivation of students at the time of promoting participation in PE.	142 students (75 girls and 67 boys, aged 12–18 years)	Quantitative methodology with a quasi-experimental non-probabilistic intra- and inter-group design with pre- and post-intervention test measures.	GAM vs. TRM	The motivation of students in PE through a gamified methodology did not entail statistically significant changes (*p* = 0.087), although a slight positive trend can be seen. In males, there is no statistically significant difference (*p* = 0.789). In females, there is a statistically significant difference (*p* < 0.045).	Significant changes in motivation with respect to GAM only occur in girls.
Ortega and Chacón-Borrego (2021)	To elaborate and develop an innovative GAM to work on alternative sports in the first year of high school. This intervention intends to offer an educational resource that helps to improve the motivation and involvement of the students in PE lessons.	111 students (12.61 ± 0.64 years, 54.95% girls and 45.05% boys, aged 11–12 years)	Non-experimental, descriptive, and longitudinal design.	Only GAM	High level of student engagement, with improvement in cooperation and respect within the classroom. GAM boosted the motivation of the students.	The use of this methodology based on the use of the “Harry Potter” universe as the main theme and of an appropriate and attractive unit design managed to improve the involvement of students, as well as learning outcomes and motivation
Quintero González et al. (2018)	To question the TRM of PE based on the use of textbooks and the standardisation of learning. To find out the students’ perception of the GAM experience, to describe the impact of ICTs’ use and the transfer of the GAM carried out and its application to other contexts	31 students (11 girls and 18 boys, aged 13–14 years)	Mixed-method study	GAM, transmedia storytelling and CL integrate in an innovative alternative “Expanded PE”	GAM enhances student motivation and engagement in PE classes. Using learning achievements as cooperative challenges within a transmedia narrative is an effective strategy to reinforce learning and extend it beyond school hours. The hybrid approach (GAM + CL) fostered awareness of pro-social behaviours such as tolerance and collaboration while providing autonomy in decision-making regarding mobile apps, SLM content, and point distribution among peers. The transmedia narrative served as a connecting thread between missions and a communication tool, allowing students to shape the experience. Student involvement was higher than in previous years.	Motivation and cooperative teaching have been reinforced with GAM and the students will work more in class.
Real-Pérez et al. (2021)	To contrast the effect of an intervention on a didactic unit applying active methodologies such as GAM, in comparison with another in which TRM was used, regarding situational motivation regarding the corporal expression contents	98 students (15.5 ± 0.537, aged 11–17 years). EXP (*n* = 49) and CON (*n* = 49)	Intervention study comparing two methodologies applied to teaching	GAM vs. TRM	There are no statistically significant differences. EXP improvement values for autonomy support, social relations support, autonomy satisfaction, intrinsic motivation, identified motivation and external motivation, decreased demotivation. CON showed improvement in terms of competence support, competence satisfaction, social relations and introjected motivation;an increase in the level of demotivation. Predisposition towards body language content. EXP improvement in terms of support for competence, satisfaction with competence, social relations, introjected motivation, skill and enjoyment. CON showed an increasing trend in the items related to effort and boredom	Innovative active methodologies seem to be an effective tool on different motivational student variables, such as: support for autonomy, support for social relationships, autonomy, intrinsic motivation, identified motivation and external motivation. Despite this, more studies are needed to determine the influence of GAM on motivation in PE lessons.
Roure and Pasco (2023)	To explore the effects of a context personalization approach through a GAM PE unit on students’ interests and perceived competence	184 students (13.9 ± 1.7, 45,1% girls and 54.9% boys, aged 11–17 years). EXP (*n* = 113) and CON (*n* = 71)	Quasi-experimental design.	GAM vs. TRM	GAM resulted in positive effects on students’ individual interest. The effects on students’ situational interest were principally moderated by students’ individual interest, indicating that the effect of the context personalization approach was higher for the students having low preintervention individual interest.	Using a context personalization approach based on a GAM unit is a promising strategy in PE to impact students’ interests, perceived competence and motivation
Segura-Robles et al. (2020)	To analyse the effects of a FL and GAM programme on the autonomy, competence, relations with others, satisfaction/enjoyment, intrinsic and extrinsic motivation, and boredom of PE students.	64 students (15 years ±1.62, 36 girls and 28 boys, aged 14–16 years)	Experimental and pre–post design based on the quantitative design	GAM and FL vs. TRM	Autonomy increased with the application of these teaching methodologies. Students’ satisfaction, enjoyment, and intrinsic motivation improved based on the interaction with GAM and FL. Academic performance also improved, although not in a significant way.	GAM and FL improved autonomy, satisfaction, enjoyment and intrinsic motivation. Academic performance also increases but not significantly.
Soriano-Pascual et al. (2022)	To compare the effects of GAM versus TRM to check whether there were differences in the attitudes of the students	66 students (33 girls and 33 boys, aged 13–16 years). EXP (*n* = 33) and CON (*n* = 33)	A quasi-experimental and longitudinal design	GAM vs. TRM	Final comparison between groups showed significant differences in all variables (*p* < 0.05) (i.e., Ego, task orientation, autonomy, relationship, irresponsibility and low commitment, disobedience, disruptive behaviour, self-control) except two [i.e., competence (*p* = 0.068) and aggressiveness (*p* = 0.136)]. In Intra-Group comparison CON showed a significant decrease in the variables task orientation (*p* = 0.004) and autonomy (*p* < 0.001). EXP all variables showed positive significant differences (*p* < 0.05), except competence (*p* = 0.223) and aggressiveness (*p* = 0.056).	With GAM, the students expressed higher levels of task orientation, all basic psychological needs and lower levels of disruptive behaviour than the students who were subjected to TRM. GAM increases motivation and decreases disruptive behaviour during PE.
Sotos-Martínez et al. (2024)	To analyse the impact of GAM on the motivation of Compulsory Secondary Education students in Spain during an 8-session Physical Education Didactic Unit	275 students (13.84 years ±1.18, 127 girls and 148 boys). EXP (*n* = 133) and CON (*n* = 142)	Quasi-experimental non-equivalent group	GAM vs. TRM	GAM improved the Basic Psychological Needs (p < 0.001) autonomy, competence, relatedness and intrinsic motivation while it decreased in amotivation (*p* = <0.001)	GAM enhances the satisfaction of the Basic Psychological Needs, increases intrinsic motivation, while it decreases a motivation.
Tenelema-Martínez and Loaiza-Dávila (2023)	To explore the application of GAM in the PE class on motivation towards learning	25 students (15.92 years ±0.49, 17 girls and 8 boys, aged 15–17 years)	A quasi-experimental design	Only GAM	Extrinsic motivation scores increased by 4.28%; intrinsic motivation increased by 5.75%, and global motivation showed an increase of almost 10%. The proportion of students with high and medium levels of motivation grew significantly.	GAM is a promising approach to reinforce motivation in the context of PE, thus enhancing students’ engagement and interest in their learning process
Valero-Valenzuela et al. (2020)	To analyse the results of a teaching intervention based on the hybridisation of the PPS with the innovative strategy of GAM	55 students (14.29 years ±0.875, 28 girls and 27 boys, aged 13–17 years)	Descriptive observational study. A mixed methodology of multilevel triangulation	Hybridisation (GAM + PPS)	Prevalence of the transfer of autonomy and responsibility in the teacher’s behaviour to the participants, which generated more self-determined motivation among the students.	The application of a programme based on the hybridisation (GAM + PPS) is effective in improving their levels of autonomy, responsibility and motivation.

CON = Control group; CL = Cooperative Learning; EXP = Experimental group; FL = Flipped learning; GAM = GAM Methodology; PE = Physical Education; PPS = pedagogical model of personal and social responsibility; PTS = Practice teaching style; SLM = Service-learning methodology; SDG = Sustainable Development Goals; TRM = Traditional Methodology; VR = Virtual reality.

Finally, Table 4 shows the benefits of Gamification and the limitations of each of the studies analyzed.

**Table 4:** Gamification benefits and study limitations.

Reference	Intervention methodology	Motivation	Academic performance	Psychological and social	Others	Limitations
Carrasco-Ramírez et al. (2019)	GAM vs. TRM	Motivation is increased using different methodologies.	Increase.			The study has no limitations.
Fernandez-Rio et al. (2021)	GAM vs. TRM	Increases the intrinsic motivation.		Increase Basic Psychological Needs, autonomy satisfaction, competence satisfaction, relatedness satisfaction.	Increase the intention to be physically active.	Small sample size. It should be borne in mind that the study was conducted in Spain. Limited intervention time. Both groups were led by the same teacher.
Fernández-Vázquez et al. (2024)	PTS vs. PTS + GAM vs. PTS + GAM + VR	Increases the motivation.			PTS group reported a higher perceived effort and a decline in the displacement with support test. All groups improvements in the handgrip strength and flamingo tests. GAM + VR increase the motor skills. GAM groups improvements lateral jump test. VR group improvements the plate-tapping tests.	Low volume of VR integrated in physical education classes (20′ per class = 240′ for the whole protocol). Different number of participants per group. Perception of effort and motor skills were not assessed.
Flores-Aguilar et al. (2023)	GAM vs. TRM	Increases the motivation.	Increase.			The study has no limitations.
Jiménez-Parra et al. (2023)	Hybridization (GAM + CL)	Increases the motivation.		Improvements in the psychological mediator’s index, executive function of planning, the PE student’s satisfaction and the school social climate, a decrease in boredom, a greater satisfaction of basic psychological needs.		Small sample size. Limited intervention time. Only the executive functions of verbal fluency and planning were analysed. Others such as cognition, social behaviour, working memory and cognitive flexibility could not be analysed. No variables related to PA level and motor engagement were measured.
Lamoneda et al. (2022)	Only GAM	Increases the motivation.		Improvements in the social relationships and personal life issues, it was fun experience, and students had allowed to disconnect and to have a sense of freedom in nature.	Learning is meaningful and promotes education in personal, social and environmental values. The holistic development of pupils is encouraged from the point of view of acquiring learning mainly in the physical, social and affective domains.	Failure to assess the sustainability of learning acquired in the long term. Absence of a control group.
López-Urán et al. (2022)	GAM vs. Attitudinal style	Increases the motivation in both groups.		In the attitudinal style model, using a task assignment style can be counterproductive, because it does not allow students to work on skills such as affective motivational.		Small sample size. Restrictions on COVID-19 measures.
Martín-Moya et al. (2018)	Only GAM	Increases the motivation.		Perception of self-perceived motor competence and perception of compared motor competence improved in the total-group. Learning commitment improved in the total-group and the male-group. Anxiety and fear of failure has increased in the total-group and the female-group.		Small sample size. Absence of a control group.
Monguillot Hernando et al. (2015)	Only GAM	Increases the motivation for learning healthy behaviours.	Increases learning.		Increases the development of healthy habits in students.	The study has no limitations.
Navalón Almedros et al. (2022)	GAM vs. TRM	Increases the motivation in female group.				Little involvement of ITCs.
Ortega and Chacón-Borrego (2021)	Only GAM	Increases the motivation.		Improve the involvement of students, cooperation and respect within the classroom.	Good learning outcomes.	The study has no limitations.
Quintero González et al. (2018)	GAM, transmedia storytelling and CL integrates in an innovative alternative “Expanded PE”	Increases the motivation.		Increase the engagement and work in PE classes. It’s reinforce learning and extend it beyond school hours. GAM + CL fostered awareness of pro-social behaviours such as tolerance and collaboration, provide autonomy in decision-making.		When using GAM, special care must be taken to achieve the stated teaching objectives.
Real-Pérez et al. (2021)	GAM vs. TRM	Increases the motivation: intrinsic motivation, identified motivation and external motivation.		An effective tool in support for autonomy and social relationships.		Limited intervention time (a didactic unit). No results are shown according to gender.
Roure and Pasco (2023)	GAM vs. TRM	Increases the motivation.		Increases the students’ interest and perceived competence.		Limited intervention time (a didactic unit). It should be noted that the study was conducted in the French part of Belgium.
Segura-Robles et al. (2020)	GAM and FL vs. TRM	Increases the intrinsic motivation.	Increases, although not in a significative way.	GAM and FL improved autonomy, satisfaction, enjoyment.		It should be noted that the study was conducted in Spain.
Soriano-Pascual et al. (2022)	GAM vs. TRM	Increases the motivation.		With the GAM, the students expressed higher levels of task orientation, all basic psychological needs, lower levels of disruptive behaviours and decrease disruptive behaviour.		Small sample size in a single educational setting. The main author, also the teacher, delivered the intervention and was not blind to the conditions.
Sotos-Martínez et al. (2024)	GAM vs. TRM	Increases the intrinsic motivation.		GAM improving the Basic Psychological Needs, autonomy, competence and relatedness while it decreases amotivation.		No qualitative measures were obtained.
Tenelema-Martínez and Loaiza-Dávila (2023)	Only GAM	Increases the extrinsic motivation, intrinsic motivation and global motivation.		Increases students’ engagement and interest in their learning process.		The study has no limitations.
Valero-Valenzuela et al. (2020)	Hybridisation (GAM + PPS)	Increases the motivation.		Increases autonomy and responsibility.		The study has no limitations.

CON = Control group; CL = Cooperative Learning; EXP = Experimental group; FL = Flipped learning; GAM = GAM Methodology; PE = Physical Education; PPS = pedagogical model of personal and social responsibility; PTS = Practice teaching style; SLM = Service-learning methodology; SDG = Sustainable Development Goals; TRM = Traditional Methodology; VR = Virtual reality.

## 4 Discussion

The main objective of this systematic review was to analyse the benefits of Gamification proposals in Physical Education in relation to motivation and meaningful learning for adolescents. To this end, a total of 19 original articles that carried out the implementation of Gamification from 2015 to 23 January 2025 were analysed.

Firstly, it could be observed that this methodology was implemented through different strategies. Some authors chose to do so without considering any other methodology (Monguillot Hernando et al., 2015; Martín-Moya et al., 2018; Ortega and Chacón-Borrego, 2021; Lamoneda et al., 2022; Tenelema-Martínez and Loaiza-Dávila, 2023). Other authors compared the effects of Gamification with a traditional teaching methodology (Carrasco-Ramírez et al., 2019; Fernandez-Rio et al., 2021; Real-Pérez et al., 2021; Navalón Almedros et al., 2022; Soriano-Pascual et al., 2022; Flores-Aguilar et al., 2023; Roure and Pasco, 2023; Sotos-Martínez et al., 2024), or with the Attitudinal Style (López-Urán et al., 2022). However, others used the hybridisation of models, combining the use of Gamification and Virtual Reality with a hands-on teaching style (Fernández-Vázquez et al., 2024), Gamification with Collaborative Learning (Quintero González et al., 2018; Jiménez-Parra et al., 2023) and Gamification with the Pedagogical Model of Personal and Social Responsibility (Valero-Valenzuela et al., 2020). Of the authors who applied hybridisation, only one compared it to traditional teaching. (i.e., Gamificación con Aprendizaje Colaborativo vs. Enseñanza Tradicional) (Segura-Robles et al., 2020).

Based on the results of the studies, it can be seen that in practically all of them there is an increase in motivation regardless of whether they have used gamification or the hybridisation of models. The only one that does not seem to show an increase in motivation is the one conducted by Carrasco-Ramírez et al. (2019), in which they pointed out that motivation did not show differences and where the effectiveness of the variation in the learning methodology was the main measure for increasing motivation. Regarding motivation, the study by Navalón Almedros et al. (2022) points to an increase in motivation only among female students. Apart from indentifying the increase in motivation, Real-Pérez et al. (2021) suggested the importance of continuing research on gamification and its effects on student motivation.

Among the most outstanding benefits, aside from motivation, some studies show benefits related to attitudes towards learning, such as autonomy (Quintero González et al., 2018; Segura-Robles et al., 2020; Valero-Valenzuela et al., 2020), the acquisition of values (Lamoneda et al., 2022) or a greater commitment to one’s own learning (Lamoneda et al., 2022; Tenelema-Martínez and Loaiza-Dávila, 2023). Others point to an increase in perceived competence (Martín-Moya et al., 2018; Roure and Pasco, 2023) and the development of healthy habits in students (Monguillot Hernando et al., 2015). Regarding the improvement of social aspects, some research highlights improvements in classroom atmosphere and a decrease in disruptive behaviour (Lamoneda et al., 2022; Soriano-Pascual et al., 2022; Jiménez-Parra et al., 2023). Finally, some show the increase and improvement of psychological needs, in some cases related to the intention to be physically active (Monguillot Hernando et al., 2015; Fernandez-Rio et al., 2021), and in others to improvements at a global level (Soriano-Pascual et al., 2022; Jiménez-Parra et al., 2023; Sotos-Martínez et al., 2024).

In terms of academic performance, there does not seem to be as homogeneous a consensus as with regard to motivation, since there are some authors who indicate an increase in overall academic performance (Carrasco-Ramírez et al., 2019; Ortega and Chacón-Borrego, 2021; Flores-Aguilar et al., 2023), whereas other studies such as Segura-Robles et al. (2020) specify that the increases in performance are not significant. Regarding the increase in specific aspects related to academic performance, Soriano-Pascual et al. (2022) suggest a higher level of task orientation and Fernández-Vázquez et al. (2024) an improvement in motor skills.

Of the studies analysed, only one shows contraindications to gamification, namely the study by López-Urán et al. (2022), which indicates that it can be counterproductive, especially when the didactic method of assigning tasks is used, because it does not allow students to work on such skills as affective-motivational ones, essential for developing the social dimension proposed by the SDGs.

Although among the studies included in this review there seems to be a consensus on the idea that gamification improves several aspects of Physical Education, it would appear that motivation is the only area where all studies indicate a betterment. Regarding other areas, there does not seem to be any agreement on which or to what an extent gamification improves aspects such as academic performance, motor skills or meaningful student learning.

Despite the findings on the relationship between the use of gamification and student motivation in physical education lessons, these should be taken with caution due to the methodological differences among the studies. Therefore, this study is not without limitations. Firstly, most of the included studies focus exclusively on the effects of gamification on student motivation without a control group using such methods as traditional or emerging ones. Therefore, very few studies make comparisons between groups. Future research should include comparative studies to better understand the differential impact of gamification on physical education. Secondly, many authors do not follow a clear methodology or do not explain it correctly in their methods section, thus there are some gaps in their explanations, making it difficult to replicate them. Finally, most are short term, which prevents the observation of the long-term effects of this methodology on physical education lessons. This temporal limitation means that the motivation, participation and physical performance of students may be temporary or vary over time and may not be contrasted with the duration of other, more long-term research.

As a main practical application, it seems that gamification is a useful tool to improve student motivation. However, there is no consensus among the different authors on improvements in other areas. It is important to interpret these results with caution due to the small sample sizes as well as the application of gamification to specific didactic units and very specific socio-demographic contexts.

In conclusion, this systematic review shows that gamification in Physical Education has a positive impact on student motivation, regardless of the way it is implemented. In addition, other benefits have been identified, such as increased autonomy, acquisition of values, improved classroom atmosphere and psychological well-being. However, there is no clear consensus on its effect on academic performance or motor skills development. These results call for further research to better understand the long-term consequences of gamification, its impact on different groups of learners and possible limitations, in order to maximise its potential for meaningful learning. Future research should focus on comparing groups (control-experimental) and monitoring whether motivation is maintained after gamification has ended. It would also be interesting to have larger sample sizes, larger contexts and longer interventions. In addition, it would be necessary to be able to measure variables related to physical activity levels and motor skills.

Based on these conclusions, gamification appears to be an effective tool for improving student motivation. However, improvements in other areas do not seem to be clearly established. In this respect, Physical Education teachers who decide to use this methodology in their sessions should bear in mind that although there is an improvement in student motivation, it may not lead to improvements in other areas such as the development of basic motor skills.

## Data Availability

The original contributions presented in the study are included in the article/supplementary material, further inquiries can be directed to the corresponding author.
